# Long non-coding RNA HoxA-AS3 interacts with EZH2 to regulate lineage commitment of mesenchymal stem cells

**DOI:** 10.18632/oncotarget.11538

**Published:** 2016-08-23

**Authors:** Xin-Xing Zhu, Ya-Wei Yan, Demeng Chen, Chun-Zhi Ai, Xifeng Lu, Shan-Shan Xu, Shan Jiang, Gen-Shen Zhong, Dong-Bao Chen, Yi-Zhou Jiang

**Affiliations:** ^1^ Institute for Advanced Study, Shenzhen University, Shenzhen, Guangdong, China; ^2^ School of Dentistry, University of California, Los Angeles, CA, USA; ^3^ Department of Physiology, Center for Diabetes, Obesity and Metabolism, Shenzhen University Health Science Center, Shenzhen University, Shenzhen, Guangdong, China; ^4^ Henan Key Laboratory of Neural Regeneration and Repairment, The First affiliated Hospital of Xinxiang Medical University, Weihui, Henan, China; ^5^ Department of Obstetrics and Gynecology, University of California, Irvine, CA, USA

**Keywords:** mesenchymal stem cells, HoxA-AS3, lineage specification, enhancer of zeste homolog 2, long non-coding RNA

## Abstract

Long non-coding RNAs (lncRNAs) play an important role in gene regulation and are involving in diverse cellular processes. However, their roles in reprogramming of gene expression profiles during lineage commitment and maturation of mesenchymal stem cells (MSCs) remain poorly understood. In the current study, we characterize the expression of a lncRNA, HoxA-AS3, during the differentiation of MSCs. We showed that HoxA-AS3 is increased upon adipogenic induction of MSCs, while HoxA-AS3 remains unaltered during osteogenic induction. Silencing of HoxA-AS3 in MSCs resulted in decreased adipogenesis and expression of adipogenic markers, PPARG, CEBPA, FABP4 and ADIPOQ. Conversely, knockdown of HoxA-AS3 expression in MSCs exhibited an enhanced osteogenesis and osteogenic markers expression, including RUNX2, SP7, COL1A1, IBSP, BGLAP and SPP1. Mechanistically, HoxA-AS3 interacts with Enhancer Of Zeste 2 (EZH2) and is required for H3 lysine-27 trimethylation (H3K27me3) of key osteogenic transcription factor Runx2. Our data reveal that HoxA-AS3 acts as an epigenetic switch that determines the lineage specification of MSC.

## INTRODUCTION

Mesenchymal Stem Cells (MSCs) have multiple differentiation potential and low immunogenicity and serve as an excellent option for regenerative medicine, tissue engineering and clinical therapy. The differentiation of MSC is regulated by specific growth factors, signaling molecules and epigenetic modifications [1, 2]. These regulatory factors together define a selective transcription of discrete combination of genes, which define a differentiation program and determine the specific lineage and phenotype. However, how specific lineage commitment is achieved and especially how epigenetic mechanisms take part in this process require further investigation.

Long non-coding RNAs (lncRNAs) are non-protein coding transcripts longer than 200 nucleotides [3]. Up to date, there are about 25,000 lncRNAs transcripts identified in the human genome. In general, the expression lncRNAs is localized in the nucleus and is more tissue-specific compared with protein-coding transcripts [4]. For example, analysis of transcriptome of mouse fat tissue revealed 175 unique lncRNAs in mature mouse adipocytes [5]. LncRNAs can bind to mRNA through base-pairing interactions, which allow RNases to degrade the mRNA [6]. LncRNAs can also be associated with transcription factors to regulate gene activation or repression. In addition, lncRNAs can function as scaffold for transcription factors and guide the transcriptional activities across the genome in *cis* or *trans* [7]. Importantly, lncRNAs are often the partners of chromatin-modifying complexes and affect gene expression. Approximately 20% of mammalian lncRNAs, including HOTAIR, bind to the polycomb repressive complex (PRC2) [4, 7]. LncRNAs are critical for various biological processes, including maintenance of stem cell pluripotency, lineage determination, cellular positional identity and metastasis of cancer cells [7–10].

Previous reports have demonstrated a link between a variety of lncRNAs and EZH2 [7, 11, 12]. EZH2 is a component of the PRC2 complex, which can repress gene transcription through catalyzing the methylation of histone 3 lysine 27 (H3K27) [13]. EZH2 plays an important role in mesenchymal lineage specification and serve as an epigenetic switch for adipogenesis and osteogenesis [14]. Moreover, depletion of lncRNA ANCR can induce osteoblast differentiation through interaction with EZH2 [12].

In current study, we found that the expression of HoxA-AS3, which locates at the HoxA loci of the genome, increased upon adipogenic induction of human MSCs (hMSCs). We hypothesized HoxA-AS3 could be a switch to direct MSCs lineage specification. We showed that silencing of HoxA-AS3 in both hMSCs and mouse MSCs (mMSCs) led to augment bone formation and reduced fat formation. HoxA-AS3 is associated with EZH2 and represses the transcription of key osteoblastic transcriptional factor RUNX2, providing new evidence for better understanding of lineage specification of MSCs through lncRNAs.

## RESULTS

### LncRNA HoxA-AS3 is required for adipogenesis of hMSCs

**Figure 1:**
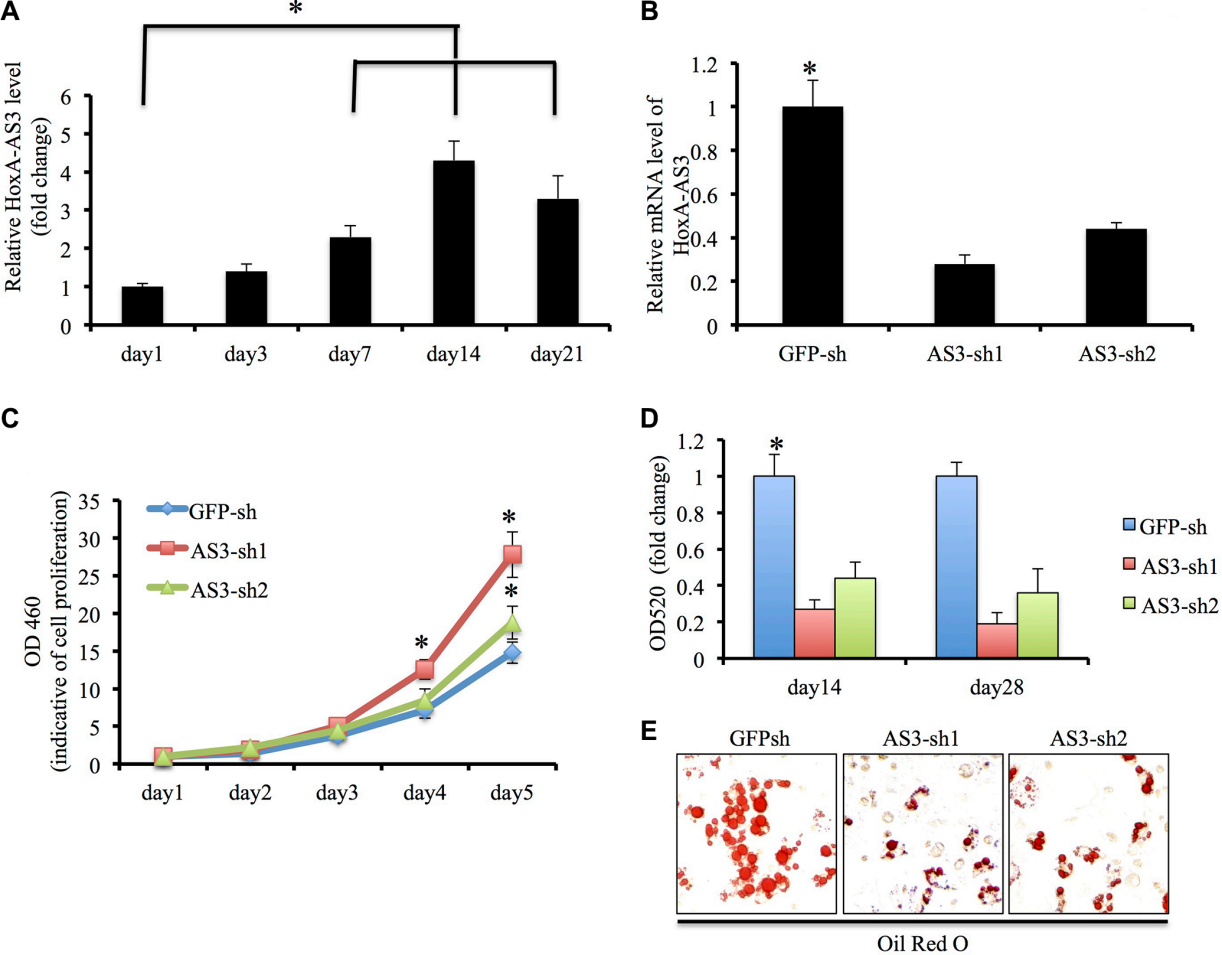
HoxA-AS3 is required adipogenesis of human MSCs (**A**) Transcript of HoxA-AS3 was upregulated in human MSCs upon adipogenic differentiation. (**B**) Human MSCs infected with HoxA-AS3-sh1 or HoxA-AS3-sh2 lentivirus reduced mRNA expression levels of HoxA-AS3 compared to control human MSCs infected with GFP-sh lentivirus (**C**) Knockdown of HoxA-AS3 promoted the proliferation of human MSCs. (**D**) Human MSCs infected with GFP-sh, HoxA-AS3-sh1 or HoxA-AS3-sh2 lentivirus were cultured in adipogenic medium for 14 and 28 days. Lipid-containing cells were stained with Oil Red O. (**E**) Quantification of Oil Red O indicated the significant lower OD520 values between HoxA-AS3 knockdown groups in comparison with control groups. (*P < 0.05).

Initially, we examined the transcript levels of HoxA-AS3 during adipogenic induction of hMSCs. Our results showed HoxA-AS3 expression was elevated 3 days onwards after adipogenic induction and reached the peak at 2 weeks (Figure 1A), suggesting that HoxA-AS3 might play a role in the adipogenesis of hMSCs. To test that possibility, we infected hMSCs with two lentivirus containing HoxA-AS3 shRNA sequences: HoxA-AS3-sh1 and HoxA-AS3-sh2. Our quantitative RT-PCR data demonstrated that both shRNA efficiently inhibited the expression of HoxA-AS3 compared with a control shRNA targeting green fluorescent protein (GFP-sh) (Figure 1B). Interestingly, we found that silencing of HoxA-AS3 stimulated proliferation of hMSCs (Figure 1C).

To determine whether depletion of HoxA-AS3 could affect adipogenic differentiation, hMSCs infected with GFP-sh, HoxA-AS3-sh1 and HoxA-AS3-sh2 lentiviral particles were cultured with adipogenic media for 28 days. As expected, hMSCs infected with HoxA-AS3-sh1 or HoxA-AS3-sh2 lentiviruses demonstrated a decreased capacity to develop Oil red O positive lipid laden adipocytes in comparison with control hMSC infected with GFP-sh lentivirus (Figure 1D).

**Figure 2:**
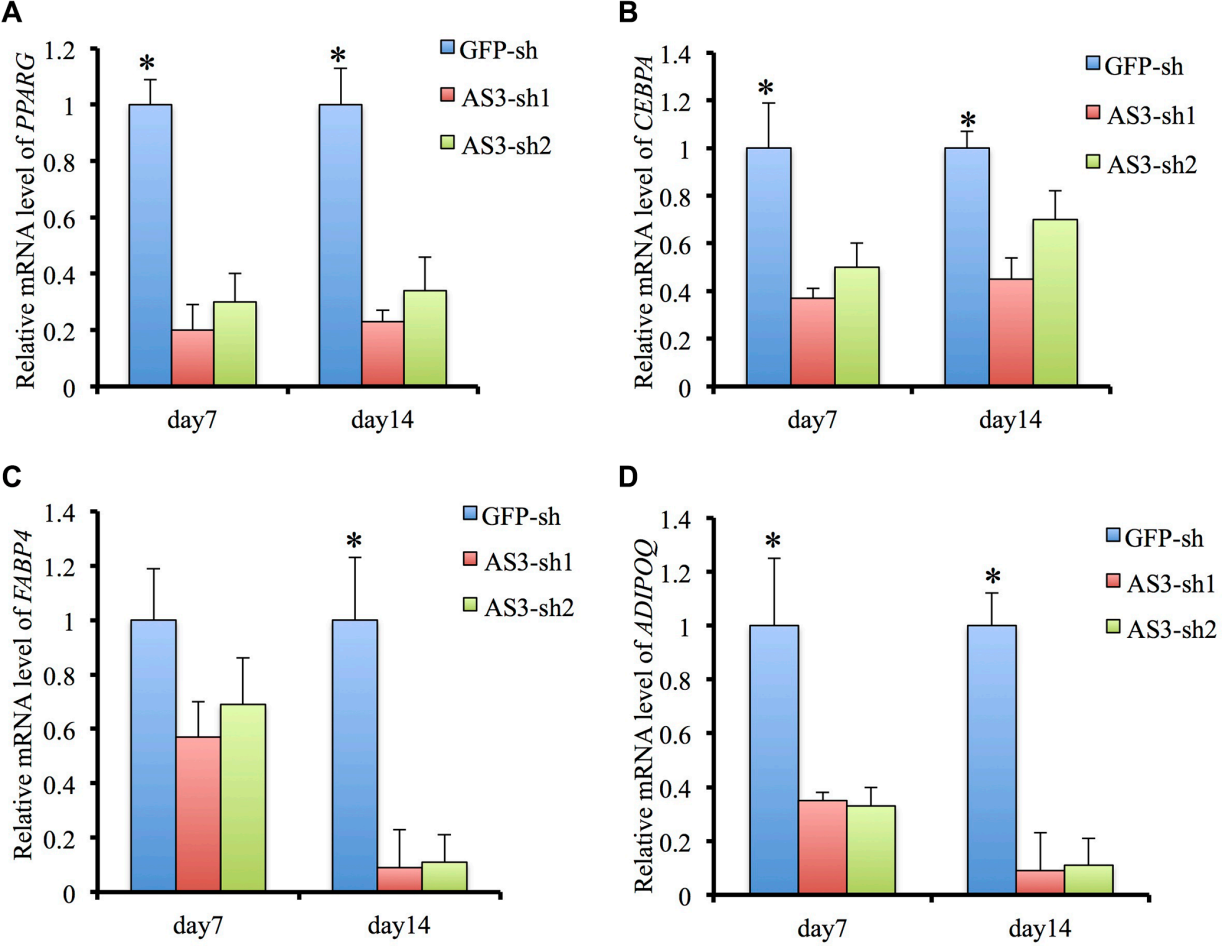
HoxA-AS3 is required for adipogenesis and adipogenic gene expression (**A**) Real-time analysis of PPARG from GFP-sh hMSCs, HoxA-AS3-sh1 and HoxA-AS3-sh2 hMSCs after 7 or 14 days of adipogenic induction. (**B**) Real-time analysis of CEBPA from GFP-sh hMSCs, HoxA-AS3-sh1 and HoxA-AS3-sh2 hMSCs after 7 or 14 days of adipogenic induction. (**C**) Real-time analysis of FABP4 from GFP-sh hMSCs, HoxA-AS3-sh1 and HoxA-AS3-sh2 hMSCs after 7 or 14 days of adipogenic induction. (**D**) Real-time analysis of ADIPOQ from GFP-sh hMSCs, HoxA-AS3-sh1 and HoxA-AS3-sh2 hMSCs after 7 or 14 days of adipogenic induction. The relative expression fold was normalized to GAPDH and calculated with ΔΔCt methods. Each experiment was performed on 3 independent samples. The data represent mean ± SD. (*P < 0.05).

Quantitative analysis confirmed that the HoxA-AS3-silencing hMSCs significantly reduced in the total amounts of Oil red O stained lipid than control hMSCs (Figure 1E). Consistently, the expressions of master transcription factors of adipogenic differentiation, *PPARγ* and *C/EBPα* were significantly decreased in HoxA-AS3-silencing hMSCs from 7 to 14 days treated with adipogenic media compared with control hMSCs (Figure 2A and 2B). Consistently, the expressions of late adipogenic markers, *FABP4* and *ADIPOQ*, were reduced dramatically in HoxA-AS3-silencing hMSCs from 7 to 14 days treated with adipogenic media compared with control hMSCs (Figure 2C and 2D). Overall, our data demonstrated that HoxA-AS3 is required for adipogenesis of MSCs.

### LncRNA HoxA-AS3 inhibits osteogenesis of hMSCs

**Figure 3:**
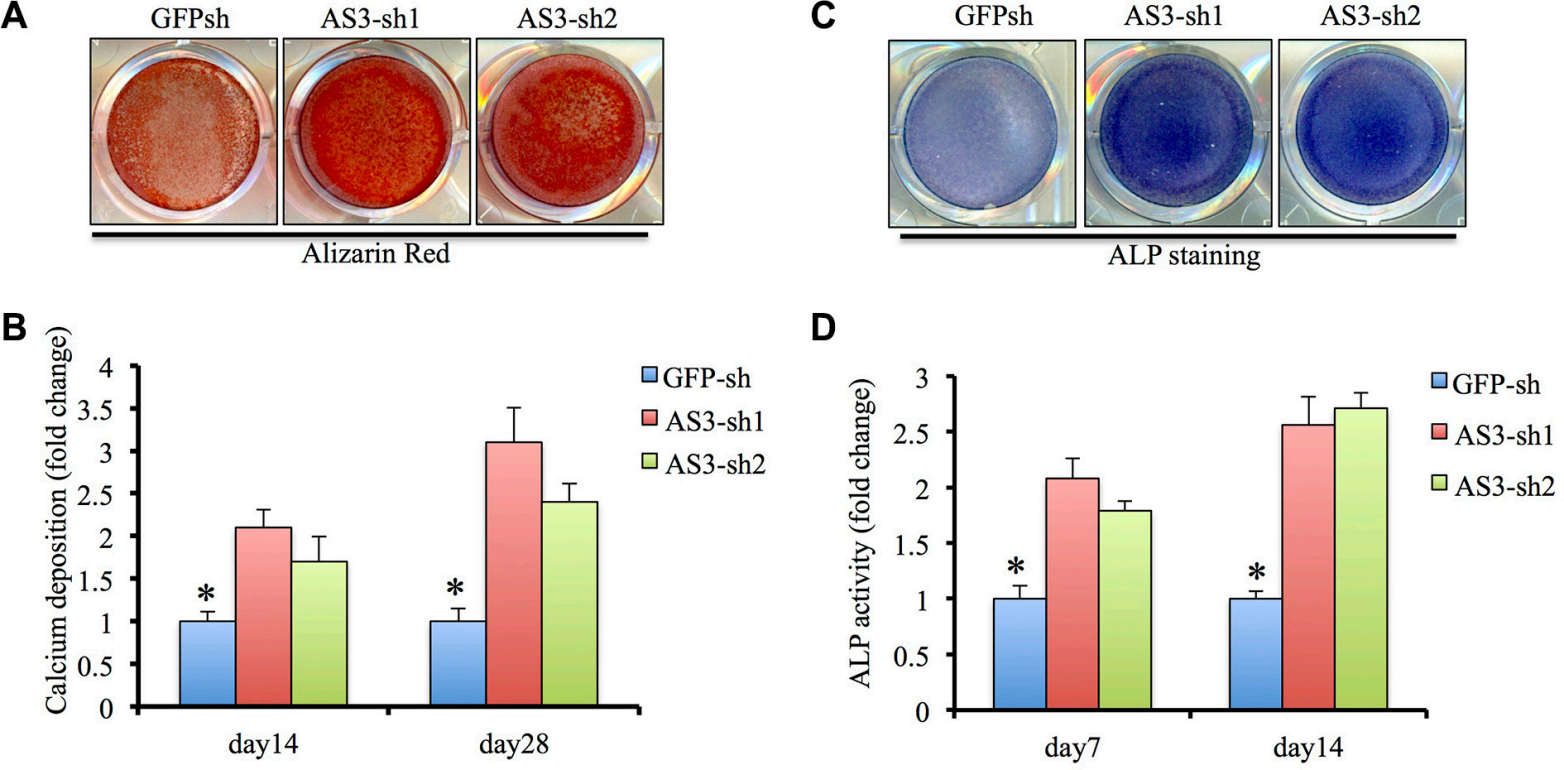
HoxA-AS3 inhibited osteogenesis of human MSCs (**A**) Alizarin Red S staining of GFP-sh hMSCs, HoxA-AS3-sh1 and HoxA-AS3-sh2 hMSCs cultured in osteogenic medium for 14 or 28 days. (**B**) Quantitative colorimetric results for ARS staining. (**C**) ALP staining of GFP-sh hMSCs, HoxA-AS3-sh1 and HoxA-AS3-sh2 hMSCs cultured in osteogenic medium for 7 or 14 days. Each experiment was performed on 3 independent samples. (**D**) Quantitative results of ALP staining. The data represent mean ± SD. (*P < 0.05).

We then endeavored to establish the function of HoxA-AS3 in hMSCs osteogenic differentiation. Functional assayed were carried out using hMSCs infected with GFP-sh, HoxA-AS3-sh1 and HoxA-AS3-sh2 lentivirus, treated in osteogenic differentiation media. Knockdown of HoxA-AS3 resulted in a dramatic increase in calcium deposition in hMSCs from 14 to 28 days by Alizarin Red staining compared with GFP-sh control cells (Figure 3A). Quantification results showed a significant induction of calcium deposition in HoxA-AS3 silencing hMSCs compared with control cells (Figure 3B). Similarly, knockdown of HoxA-AS3 caused increased alkaline phosphatase (ALP) activity at 14 days treated with osteogenic media compared with control hMSCs (Figure 3C and 3D).

**Figure 4:**
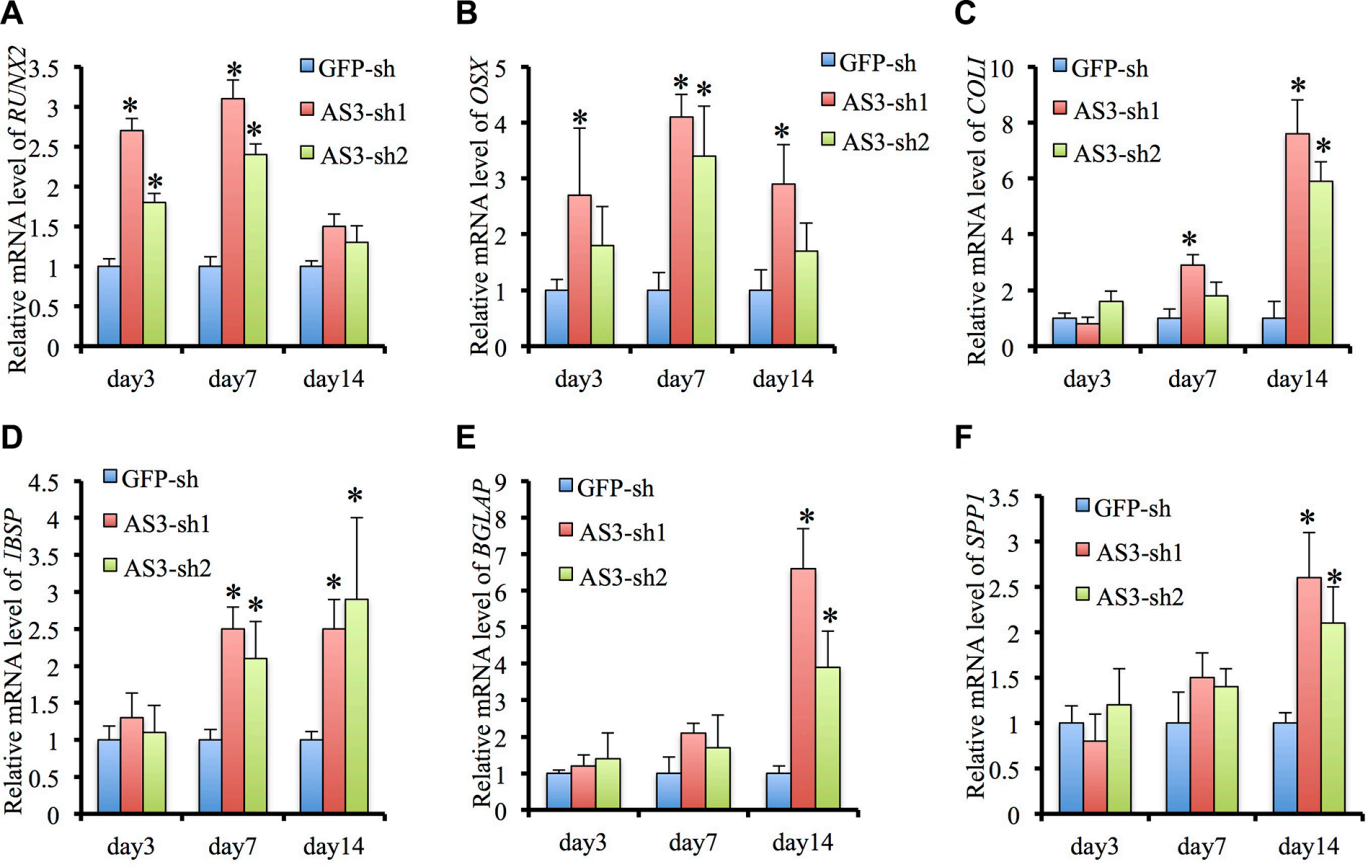
HoxA-AS3 inhibited osteogenic gene expression of human MSCs (**A**) Real-time analysis of RUNX2 from GFP-sh hMSCs, HoxA-AS3-sh1 and HoxA-AS3-sh2 hMSCs after 3, 7 or 14 days of osteogenic induction. (**B**) Real-time analysis of SP7 from GFP-sh hMSCs, HoxA-AS3-sh1 and HoxA-AS3-sh2 hMSCs after 3, 7 or 14 days of osteogenic induction. (**C**) Real-time analysis of COL1A1 from GFP-sh hMSCs, HoxA-AS3-sh1 and HoxA-AS3-sh2 hMSCs after 3, 7 or 14 days of osteogenic induction. (**D**) Real-time analysis of IBSP from GFP-sh hMSCs, HoxA-AS3-sh1 and HoxA-AS3-sh2 hMSCs after 3, 7 or 14 days of osteogenic induction. (**E**) Real-time analysis of BGLAP from GFP-sh hMSCs, HoxA-AS3-sh1 and HoxA-AS3-sh2 hMSCs after 3, 7 or 14 days of osteogenic induction. (**F**) Real-time analysis of SPP1 from GFP-sh hMSCs, HoxA-AS3-sh1 and HoxA-AS3-sh2 hMSCs after 3, 7 or 14 days of osteogenic induction. The relative expression fold was normalized to GAPDH and calculated with ΔΔCt methods. Each experiment was performed on 3 independent samples. The data represent mean ± SD. (*P < 0.05).

Quantitative RT-PCR analysis of HoxA-AS3-silencing hMSCs displayed a dramatic induction in expression of osteogenic master transcription factors, RUNX2 and SP7, and the mature bone associated markers, COL1A1, IBSP, BGLAP and SPP1, compared with GFP-sh hMSCs, following osteogenic induction (Figure 4A–4F). Collectively, our data demonstrate that HoxA-AS3 is required for inhibition of hMSC osteogenic differentiation.

### Knockdown of LncRNA HoxA-AS3 promotes osteogenesis of hMSCs *in vivo*

**Figure 5:**
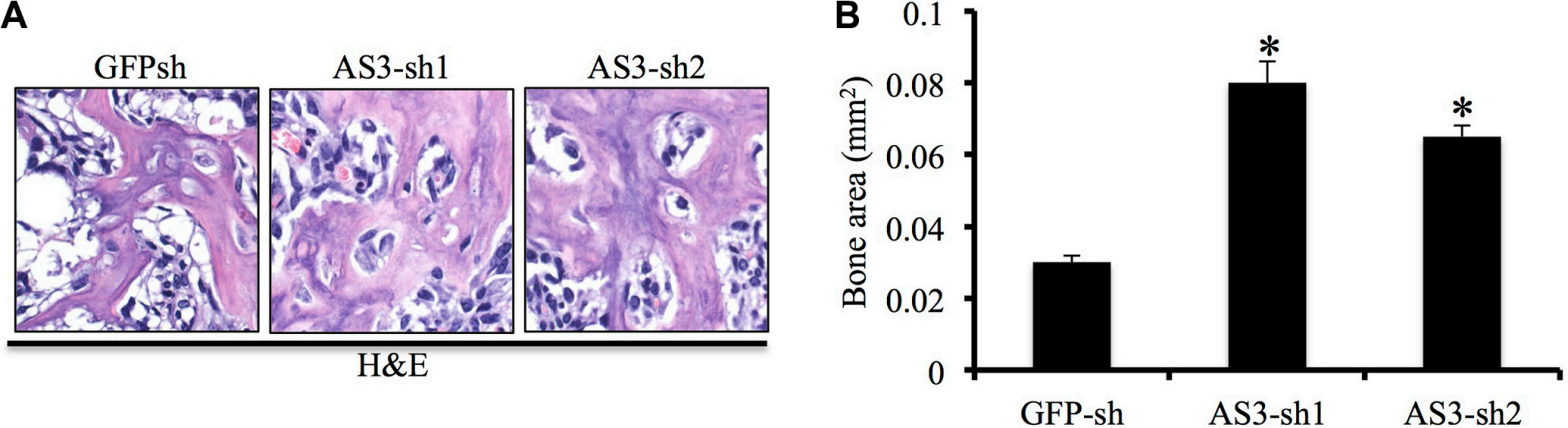
Depletion of HoxA-AS3 promotes hMSCs-mediated heterotopic bone formation *in vivo* (**A**) Routine H&E staining shows that the tissues generated *in vivo* contain more bone tissue in HoxA-AS3-silengcing hMSCs. (**B**) Corresponding quantitative data shows that significant difference in the total area of bone tissue between control and HoxA-AS3-silencing groups. The data represent mean ± SD. (*P < 0.05).

To confirm whether silencing of HoxA-AS3 supported hMSC-mediated bone formation *in vivo*, we injected hMSCs infected with GFP-sh, HoxA-AS3-sh1 and HoxA-AS3-sh2 combined tricalcium phosphate/hydroxyapatite (TCP/HA) scaffolds subcutaneously into nude mice. Heterotopic bones were then collected after 2 months for analysis histologically. We observed increased formation of bone in mice injected with HoxA-AS3-sh1 and HoxA-AS3-sh2 hMSCs compared with GFP-sh hMSCs (Figure 5A). Our quantification results revealed 2.6-fold and 2.2-fold increase bone formation in HoxA-AS3-sh1 and HoxA-AS3-sh2 hMSCs groups, respectively, than the control group (Figure 5B). Taken together, we findings showed silencing of HoxA-AS3 can promote osteogenesis of hMSCs both *in vitro* and *in vivo*.

### LncRNA HoxA-AS3 promotes adipogenesis and inhibits osteogenesis of mMSCs

To verify the human finding, we also generated two HoxA-AS3-sh mMSCs lines. Functional studies showed that silencing of HoxA-AS3 in mMSCs resulted in a dramatic decrease in Oil Red O positive lipid producing adipocytes under adipogenic condition compared with GFP-sh control mMSCs (Supplementary Figure S1A). Consistently, silencing of HoxA-AS3 in mMSCs displayed increased ALP activities and mineral deposition over control mMSCs in osteogenic media (Supplementary Figure S1B and S1C), indicating a conservative role of HoxA-AS3 in mediating lineage specification of MSC across different mammalian species.

### LncRNA HoxA-AS3 binds to EZH2 and regulates *RUNX2* expression

**Figure 6:**
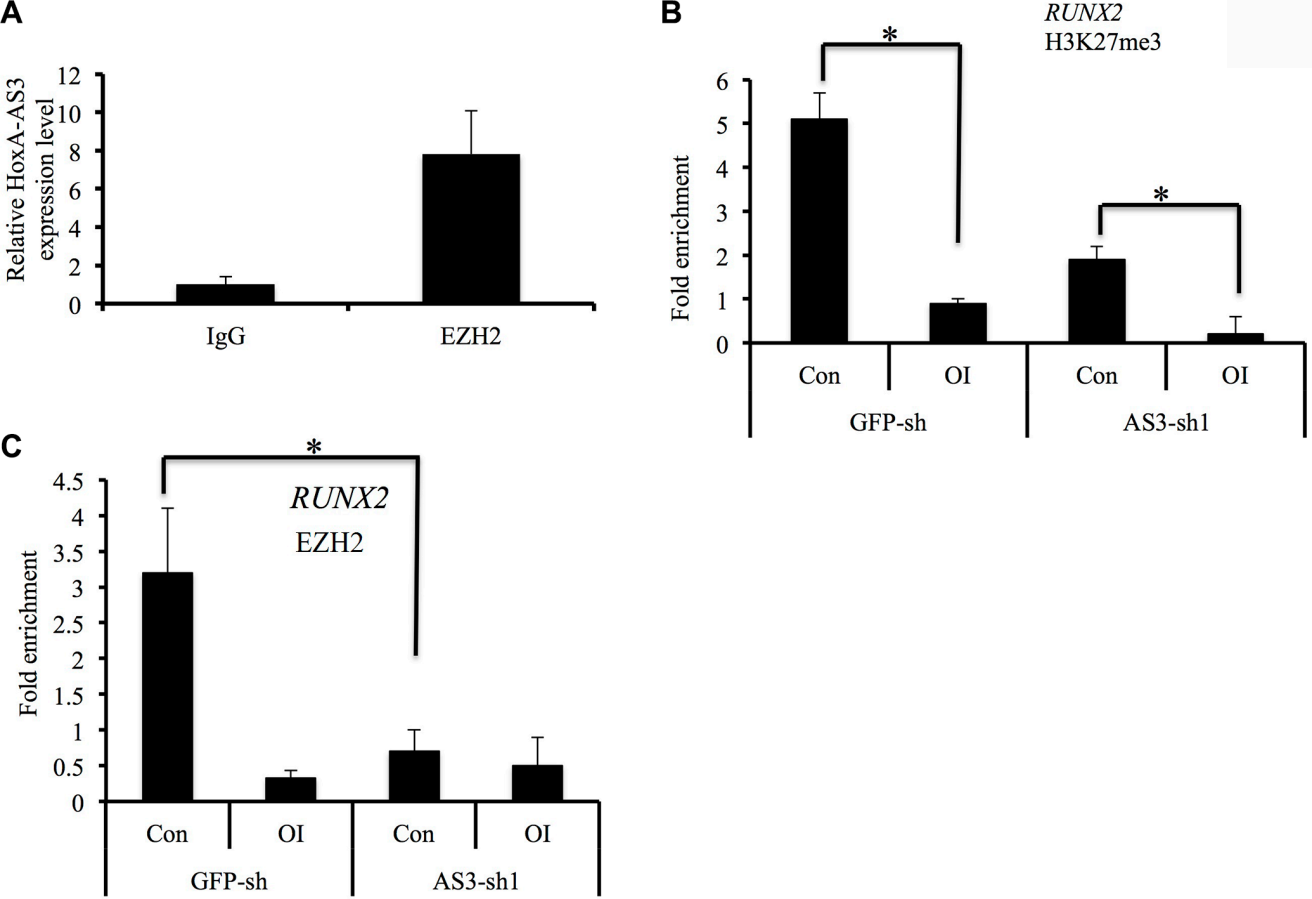
HoxA-AS3 is associated with EZH2 and regulates *RUNX2* expression (**A**) Immunoprecipitation of EZH2 retrieves endogenous HoxA-AS3. (**B, C**) ChIP-PCR analysis was conducted for the Runx2 promoter regions of HoxA-AS3-silencing or control hMSCs using H3K27me3 (B) and EZH2 (C) antibodies with or without osteogenic induction (OI). Relative enrichment to the input controls was calculated and shown. Data presented were average of three independent experiments expressed as the mean ± SD. *p < 0.05.

Since lncRNAs are often associated with components of polycomb repressive complex 2 (PRC2), we next assessed the binding activity between HoxA-AS3 and EZH2. Indeed, native immunoprecipitation of EZH2 from nuclear extracts of hMSCs retrieved 7.8-fold enrichment of endogenous HoxA-AS3 as detected by qRT-PCR compared with IgG control (Figure 6A), suggesting that HoxA-AS3 may play a role in EZH2-dependent gene repression.

To test that, we first examined the levels of H3K27me3, which is catalyzed by EZH2, in control and HoxA-AS3-sh1 hMSCs after osteogenic induction. Our ChIP-PCR data showed that the H3K27me3 level at RUNX2 promoter region was significantly decreased 3 days after treatment of osteogenic media in control hMSCs, suggesting a derepression of RUNX2 gene (Figure 6B). Importantly, the silencing of HoxA-AS3 led to a further decrease of H3K27me3 level at RUNX2 promoter region of hMSCs under control condition (Figure 6B), indicating a role of HoxA-AS3 in EZH2-mediated gene repression. To explore whether HoxA-AS3 is required for occupation of EZH2 at chromatin region, we carried out EZH2 ChIP-PCR experiment. We found that depletion of HoxA-AS3 led to 4.5 fold reduce of EZH2 binding to the promoter region of RUNX2 gene (Figure 6C), demonstrating that HoxA-AS3 is associated with EZH2 in regulating transcription repression activities.

## DISCUSSION

MSCs-based regenerative medicine is now one of the fastest developing fields in biology, which provides promising treatments for a variety of human diseases. Thus, identifying factors capable of enhancing differentiation efficiency of MSCs seems to be essential when application of MSCs in the field of regenerative medicine is considered. We found that the expression of a specific lncRNA, HoxA-AS3 in hMSCs under adipogenic condition. By using virus-mediated silencing of HoxA-AS3 in both hMSCs and mMSCs, we found this led to increase of bone formation and decrease of fat formation. In addition, knockdown of HoxA-AS3 led to increase of MSC proliferation, which holds a promising potential for better regeneration of bone tissue. We further showed that HoxA-AS3 could be associated with EZH2 and represses RUNX2 expression. Our study is the first study that characterizes the role of lncRNA HoxA-AS3 in mediating MSCs lineage determination, providing the evidence for HoxA-AS3 as an important target for guiding more efficient design for MSC-based therapy.

Upon adipogenic induction, we found that HoxA-AS3 expression was increased in a time-dependent manner. By analyzing the transcriptome of primary brown and white adipocytes, preadipocytes, and cultured adipocytes, researcher have previously identified 175 unique lncRNAs that were essential for adipogenesis [5]. Functionally, lncRNA can regulate the expression of key transcription factor for adipogenesis. For example, lncRNA ADINR could promote adipogenesis by activating C/EBPα transcription [15]. Alternatively, PU.1 antisense lncRNA promotes adipogenesis by counteracting mRNA translation of PU.1 [16], indicating a versatile role of lncRNA during adipogenesis. The exact molecular mechanism of how HoxA-AS3 is required for adipogenic differentiaton of MSC remains unknown. However, based on the fact that silencing of HoxA-AS3 led to decrease of PPARγ and C/EBPα, it seems like HoxA-AS3 might have a role in transcription regulation in MSCs.

Previous report showed that lncRNA MEGs could induce osteogenic differentiation and expression of RUNX2 of MSC through targeting the transcription of BMP4 [17]. Another study showed lncRNA H19 promotes osteogenesis by acting as a miRNA sponge and competing with endogenous RNA [18]. We showed that HoxA-AS3 regulates osteogenesis through derepression of RUNX2. Since HoxA-AS3 affect both adipogenic and osteogenic lineage, we set up to investigate those master transcription factors in lineage determination, including Runx2, Sp7, CEBP/α, PPARγ. However, we can only detect direct binding of HoxA-AS3 to Runx2 promoter using ChIP approach. This doesn't exclude the possibility that HoxA-AS3 can bind to other master transcription factors. To further explore the detailed mechanism of how HoxA-AS3 in regulating MSC lineage specification, a RIP-Seq will need to be performed. Still, our result of Runx2 repression by HoxA-AS3 can explained our finding. Runx2 not only serves as a transcription factor for osteogenic specification, but also acts as repressor of adipogenesis of MSC [19].

HoxA-AS3 is associated with a PCR2 component, EZH2. However, which portion of HoxA-AS3 is responsible for this interaction has yet to be determined. LncRNAs frequently bind to the core components of chromatin-modifying complexes, such as EZH2 and SUZ12 and regulate gene expression [4, 7]. Interestingly, a previous report showed inhibition of EZH2 activity by chemical inhibitor or depletion of Ezh2 gene expression by siRNA in hMSC can cause reduction of adipogenesis and induction of osteogenesis [14], in consistent with our current finding. How does depletion of HoxA-AS3 and EZH2 affect the global gene expression in MSCs and whether they can co-occupy the same domain of chromatin globally need further investigation.

In summary, this study has identified an epigenetic player, HoxA-AS3, involving EZH2-dependent H3K27me3 modification and the lineage specification of MSCs into adipocytes and osteoblasts. Understanding the molecular mechanisms of lncRNAs in regulating MSC lineage determination is essential for understanding MSC-related diseases, such as bone aging and osteoporosis.

## MATERIALS AND METHODS

### Cell culture

Human mesenchymal stem cells-bone marrow was obtained from ScienCell^TM^ Research Laboratories. Mouse mesenchymal stem cells (mMSC) were purchased from GIBCO (C57BL/6 Mouse Mesenchymal Stem Cells). Both human and mouse MSCs were cultured in Dulbecco's modified Eagle's medium (DMEM) containing 10% fetal bovine serum (FBS, Hyclone Laboratories, China), 100 IU/ml penicillin and 100 μg/ml streptomycin (Gibco, China), 2 mM l-glutamine (Gibco, China) and 1% non-essential amino acids (NEAA; Sigma-Aldrich, China) at 37°C in a humidified atmosphere of 5% CO_2_ in air.

### Plasmids construction and lentiviral infection

For HoxA-AS3 silencing experiments, 2 pairs of HoxA-AS3-shRNA against human or mouse and a control GFP-shRNA were designed and cloned into the modified pLV-H1-Puro lentiviral vector [20]. The corresponding sequences were: HoxA-AS3-sh1, 5′-AGCCAGGTTGCGAGTTGCAAA-3′ (for human), HoxA-AS3-sh2, 5′-AAGGGCCGAACAACTCATAAA-3′ (for human), HoxA-AS3-sh3, 5′-AGCCAGGTTGCA AGTTGCAAA-3′ (for mice), HoxA-AS3-sh4, 5′-AAGGG CCGAACAACTCATAAA-3′ (for mice), and GFP-sh, 5′-TACAACAGCCACAACGTCTAT-3′. Lentiviruses carrying these vectors were produced in a HEK293T cell line by transient transfection with Lipofectamin2000 (invitogen, China). Cell supernatants containing lentiviral particles were collected after transfection for 48 h. For transduction, 1 × 10^5^ hMSCs or mMSCs were seeded into 6-well plates and incubated with lentiviral particles together with 8 μg/ml polybrene in the incubator for 16 h. Stable infected MSCs were then established in the presence of puromycin (20 μg/ml) for 10 days. The expression of HoxA-AS3 in the infected stable MSCs was examined using quantitative RT-PCR. The primer sequences for were:

HoxA-AS3 Forward 5′-CACCTCTCTCATCGAA AAACCG-3′,

HoxA-AS3 Reverse 5′- GCACCAGGAAAGAG GACAATTC-3′;

GAPDH Forward 5′-TCGCTCCTGGAAGATG GTGAT-3′;

GAPDH Reverse 5′-TCATTGACCTCAACTAC ATG-3′.

### Cell proliferation assays

A cell proliferation assay was performed with MTT kit (Sigma, China) as previously described [21]. Cells were placed into 96-well plate and maintained in media containing 10% FBS for 5 days. Cell proliferation was measured every day for 6 days using the manufacturer's guidance.

### Osteogenic differentiation of MSCs

To induce osteoblast differentiation of hMSCs or mMSCs, the culture medium was removed after cell reach confluency and replaced by osteogenic medium containing α-MEM with 10% FBS, 10 mM β-glycerol phosphate, 10 nM dexamethasone and 50 μg/ml ascorbic acid phosphate (Sigma-Aldrich, China). The medium was changed every 3 days for 2–4 weeks. At the end point of the assays, Alizarin-Red staining (Sigma, China) was used to detect the calcium deposition associated with osteoblasts. For Alizarin-Red staining, cells were fixed in 70% ethanol for 30 min and then subjected to 1% Alizarin-Red solution for 1 min. Images of the stained cells were scanned and captured. Alizarin Red staining were then dissolved using a solution of 20% methanol and 10% acetic acid in water and quantified by measuring its absorbance at 450 nm. Relative Alizarin Red staining was then calculated as a fold change of the control. For ALP staining, cells were fixed with 70% ethanol for 30 min and then incubated with the BCIP/NBT liquid substrate system (Sigma-Aldrich, China) at 37°C for 30 min. Images of the stained cells were scanned and captured. For quantitative analysis, the ALP stain was extracted with 10% cetylpyridinium chloride for 15 min and quantified by measuring its absorbance at 540 nm. Relative ALP staining was then calculated as a fold change of the control.

### Adipogenic differentiation of MSCs

For adipogenic induction, MSCs after reaching confluency was cultured using adipogenic differentiation medium consisted of α-MEM supplemented with 10% FBS, 100 nM dexamethasone, and 50 mg/ml indomethacin (Sigma, China). The culture medium was changed every 3 days for 3 weeks. For Oil-Red O (Sigma, China) staining, the cultures were fixed with 70% ethanol for 30 min, and then stained with Oil-Red O for 15 min. The cells were then maintained in PBS for light-microscope imaging. To quantify staining, Oil Red-O was extracted from cells on the slides with isopropanol containing 4% Nonidet P-40, and optical density was then measured at a wavelength of 520 nm. Relative Oil-Red O staining was then calculated as a fold change of the control.

### Quantitative RT-PCR

RNA was extracted from tissue samples using TRIzol reagent (Invitrogen, China) as previously described [22, 23]. Contaminated genomic DNA was removed by DNAase I treatment (Invitrogen, China). For qRT-PCR, RNA was reverse transcribed to cDNA by using a Reverse Transcription Kit (Takara, Dalian, China). Real-time PCR analyses were performed with Power SYBR Green (Takara, Dalian China) and the primers for each gene were designed as previously described [24]. Results were normalized to the expression of GAPDH. The relative difference in the expression level was calculated using the ^ΔΔ^CT method. The data presented are representative of three independent biological repeats each assayed in triplicate and are relative expression levels.

### Mouse transplantation and histology analysis

All animal-related procedures were preapproved by the institutional ethics committee.

The cells were trypsinized to a single-cell suspension and seeded onto TCP/HA were then implanted subcutaneously in the dorso-lateral area of 10 weeks old BALB/c nude mice (Model Animal Research Center, Nanjing, China) for 8 weeks. The ectopic bone were collected, fixed in formalin, decalcified in ethylene diamine tetraacetic acid (EDTA) for 12 days, embedded, and sectioned. H&E were performed followed the protocol as previous described [1].

### RNA binding protein immunoprecipitation (RIP) assay

RNA immunoprecipitation was performed using the EZ-Magna RIP kit (Millipore, China) following the manufacturer's guidance. 2 × 10^7^ hMSCs at 80–90% confluency were lysed in 2 ml PBS, 2 ml nuclear isolation buffer (1.28 M sucrose; 40 mM Tris-HCl pH 7.5; 20 mM MgCl2; 4% Triton X-100) and 6 ml ddH_2_O. Nuclei was resuspended in RIP buffer, sonicated and incubated with magnetic beads conjugated with human anti-EZH2 antibody (Abcam, China), negative control normal mouse IgG (Abcam, China) overnight followed by stringent washing of protein A/G bead pellets. Samples were incubated with Proteinase K with shaking to digest the protein and then immunoprecipitated RNA was isolated. Furthermore, purified RNA was subjected to qRT-PCR analysis to demonstrate the presence of HoxA-AS3.

### ChIP assays

ChIP assays were carried out followed a protocol as previously described [21]. For each ChIP reaction, 2 × 10^6^ hMSCs were fixed in formaldehyde for 15 min in a 37°C water-bath. Cross-linked chromatin was sonicated to generate 200–500 bp fragments. The chromatin was immunoprecipitated using anti-EZH2 or anti-H3K27me3 antibody (Abcam, China). Normal human IgG was used as a negative control. All resulting precipitated DNA samples were quantified with a specific set of primers for individual genes using real-time PCR as previously described [12].

### Statistics

Data analysis, graph generation, and statistical analysis were carried out using the Microsoft office software. Paired or unpaired Student's *t*-test were used for differentiation and gene expression analysis and quantification of Alizarin Red, ALP, Oil Red O staining. Statistical differences (*) of *P* < 0.05 between samples are shown.

## SUPPLEMENTARY MATERIALS FIGURES


