# Correction: Long non-coding RNA HoxA-AS3 interacts with EZH2 to regulate lineage commitment of mesenchymal stem cells

**DOI:** 10.18632/oncotarget.25078

**Published:** 2018-04-03

**Authors:** Xin-Xing Zhu, Ya-Wei Yan, Demeng Chen, Chun-Zhi Ai, Xifeng Lu, Shan-Shan Xu, Shan Jiang, Gen-Shen Zhong, Dong-Bao Chen, Yi-Zhou Jiang

**Affiliations:** ^1^ Institute for Advanced Study, Shenzhen University, Shenzhen, Guangdong, China; ^2^ Key Laboratory of Optoelectronic Devices and Systems of Ministry of Education and Guangdong, College of Optoelectronic Engineering, Shenzhen University, Shenzhen, Guangdong, China; ^3^ School of Dentistry, University of California, Los Angeles, CA, USA; ^4^ Department of Physiology, Center for Diabetes, Obesity and Metabolism, Shenzhen University Health Science Center, Shenzhen University, Shenzhen, Guangdong, China; ^5^ Henan Key Laboratory of Neural Regeneration and Repairment, The First affiliated Hospital of Xinxiang Medical University, Weihui, Henan, China; ^6^ Department of Obstetrics and Gynecology, University of California, Irvine, CA, USA

**This article has been corrected:** The correct author affiliations are given below:

**Xin-Xing Zhu**^1,2,*^, Ya-Wei Yan^1,2,*^, Demeng Chen^**3**^**, Chun-Zhi Ai**^**1**^**, Xifeng Lu**^**4**^**, Shan-Shan Xu**^**1**^**, Shan Jiang**^**1**^**, Gen-Shen Zhong**^**5**^**, Dong-Bao Chen**^**6**^ **and Yi-Zhou Jiang**^**1**^

^**1**^ Institute for Advanced Study, Shenzhen University, Shenzhen, Guangdong, China

^**2**^ Key Laboratory of Optoelectronic Devices and Systems of Ministry of Education and Guangdong, College of Optoelectronic Engineering, Shenzhen University, Shenzhen, Guangdong, China

^**3**^ School of Dentistry, University of California, Los Angeles, CA, USA

^**4**^ Department of Physiology, Center for Diabetes, Obesity and Metabolism, Shenzhen University Health Science Center, Shenzhen University, Shenzhen, Guangdong, China

^**5**^ Henan Key Laboratory of Neural Regeneration and Repairment, The First affiliated Hospital of Xinxiang Medical University, Weihui, Henan, China

^**6**^ Department of Obstetrics and Gynecology, University of California, Irvine, CA, USA

^*^ These authors contributed equally to this work

Original article: Oncotarget. 2016; 7:63561-63570. https://doi.org/10.18632/oncotarget.11538

