# Supplementary material for: Long non-coding RNA HoxA-AS3 interacts with EZH2 to regulate lineage commitment of mesenchymal stem cells
Source: Oncotarget. 2016 Aug 23;7(39):63561–70. doi: 10.18632/oncotarget.11538 (PMC5325385; doi:10.18632/oncotarget.11538)
Supplement: Supplementary file 1 [file oncotarget-07-63561-s001.pdf]

Long non-coding RNA HoxA-AS3 interacts with EZH2 to regulate lineage commitment of mesenchymal stem cells

Supplementary Materials

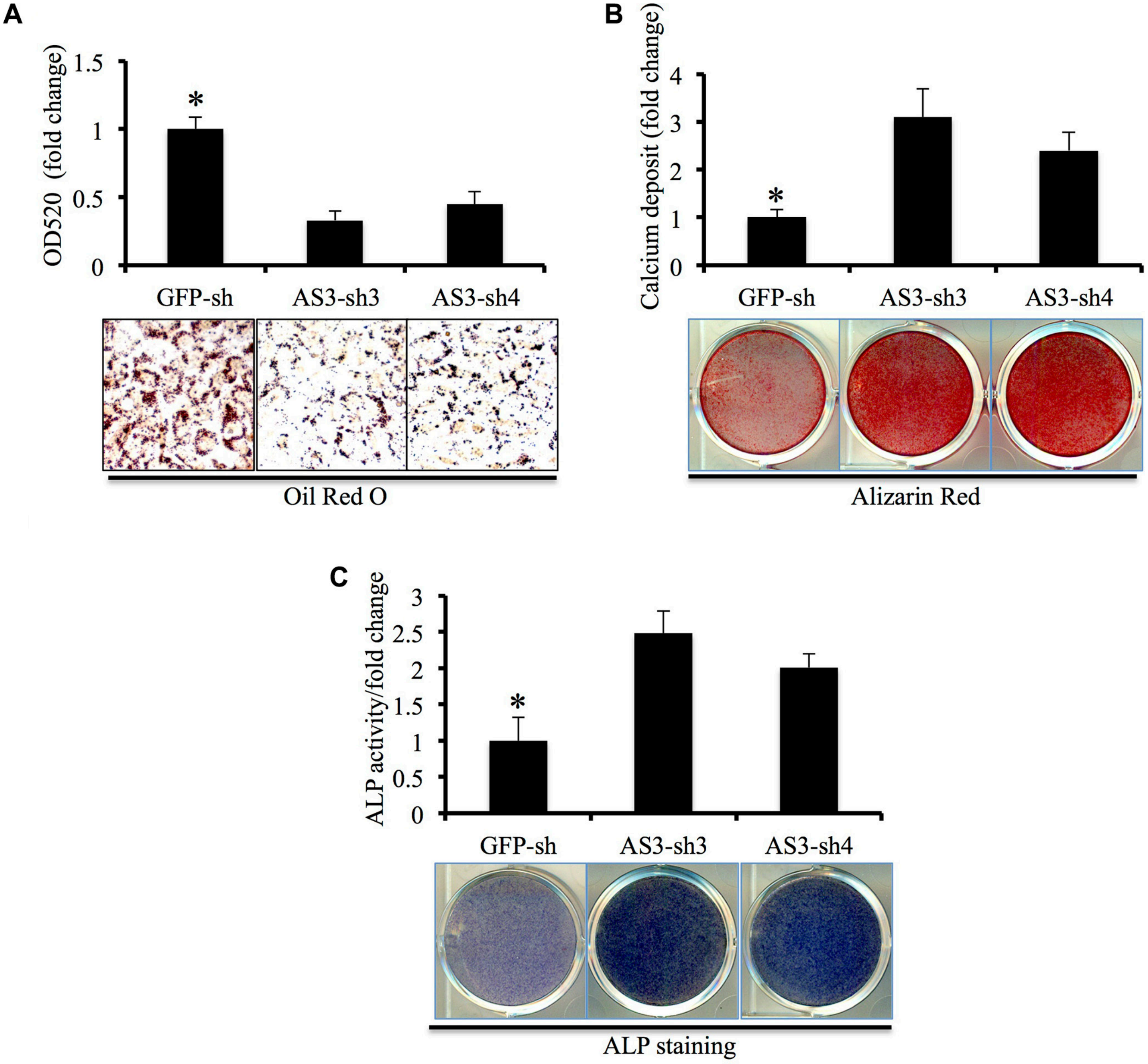

Supplementary Figure S1: HoxA-AS3 promotes adipogenesis and inhibits osteogenesis of mMSCs. (**A**) Mouse MSCs infected with GFP-sh, HoxA-AS3-sh3 and HOXA-AS3-sh4 lentivirus were cultured in adipogenic medium and lipid-containing cells were stained with Oil Red O. Quantification of Oil Red O indicated the significant lower OD520 values between HoxA-AS3 knockdown groups in comparison with control groups. (**B**) Alizarin Red S staining and quantitative colorimetric results of GFP-sh mMSCs, HoxA-AS3-sh3 and HOXA-AS3-sh4 mMSCs cultured in osteogenic medium for 28 days. (**C**) ALP staining and quantitative results of GFP-sh mMSCs, HoxA-AS3-sh3 and HOXA-AS3-sh4 mMSCs cultured in osteogenic medium for 28 days. Each experiment was performed on 3 independent samples. The data represent mean ± SD. (*$P < 0.05$).
